# Preanalytic and analytic factors affecting the measurement of haemoglobin concentration: impact on global estimates of anaemia prevalence

**DOI:** 10.1136/bmjgh-2021-005756

**Published:** 2021-07-30

**Authors:** Leila M Larson, Sabine Braat, Mohammed Imrul Hasan, Martin N Mwangi, Fernando Estepa, Sheikh Jamal Hossain, Danielle Clucas, Beverley-Ann Biggs, Kamija S Phiri, Jena Derakhshani Hamadani, Sant-Rayn Pasricha

**Affiliations:** 1 Department of Health Promotion, Education, and Behavior, University of South Carolina, Columbia, South Carolina, USA; 2 Centre for Epidemiology and Biostatistics, The University of Melbourne, Melbourne, Victoria, Australia; 3 Department of Infectious Diseases at the Peter Doherty Institute, The University of Melbourne, Melbourne, Victoria, Australia; 4 Population Health and Immunity Division, Walter and Eliza Hall Institute of Medical Research, Melbourne, Victoria, Australia; 5 Maternal and Child Health Division, International Centre for Diarrhoeal Disease Research Bangladesh, Dhaka, Bangladesh; 6 School of Public Health and Family Medicine, University of Malawi College of Medicine, Blantyre, Malawi; 7 The Royal College of Pathologists of Australasia Quality Assurance Program, Melbourne, Victoria, Australia; 8 Diagnostic Haematology, The Royal Melbourne Hospital, Melbourne, Victoria, Australia; 9 Clinical Haematology, The Peter MacCallum Cancer Centre and The Royal Melbourne Hospital, Melbourne, VIC, Australia; 10 The Victorian Infectious Diseases Service, The Royal Melbourne Hospital, Melbourne, Victoria, Australia

**Keywords:** anaemia, diagnostics and tools

## Abstract

The accuracy of haemoglobin concentration measurements is crucial for deriving global anaemia prevalence estimates and monitoring anaemia reduction strategies. In this analysis, we examined and quantified the factors affecting preanalytic and analytic variation in haemoglobin concentrations. Using cross-sectional data from three field studies (in children, pregnant and nonpregnant women), we examined the difference in haemoglobin concentration between venous-drawn and capillary-drawn blood measured by HemoCue (ie, preanalytic) and modelled how the bias observed may affect anaemia prevalence estimates in population surveys and anaemia public health severity classification across countries. Using data from an international quality assurance programme, we examined differences due to instrumentation from 16 different haematology analyzers (ie, analytic). Results indicated that capillary and venous haemoglobin concentrations are not in agreement (bias +5.7 g/L (limits of agreement (LoA) −11.2, 22.6) in preschool age children; range from −28 g/L to +20 g/L in pregnant women; bias +8.8 g/L (LoA −5.2, 22.9) in non-pregnant women). The bias observed could introduce changes in population survey estimates of anaemia of up to −20.7 percentage points in children and −28.2 percentage points in non-pregnant women after venous adjustment. Analytic variation was minimal and unlikely to influence the diagnosis of anaemia. These findings suggest that global estimates of anaemia prevalence derived from capillary haemoglobin, as they often are, may be inaccurate and lead to erroneous public health severity classification, but that point-of-care, or other, instruments should not introduce variation if properly used.

**Key questions**

**What is already known?**

- Limited evidence suggests that there may be variation in haemoglobin concentration measured in population-based surveys using field-suitable techniques and the true prevalence of anaemia may be under or overestimated.

**What are the new findings?**

- Variation in venous haemoglobin concentrations was minimal when measured using 16 different commercial haematology analyzers and point-of-care devices.
- Capillary and venous haemoglobin concentrations are not in agreement in preschool age children, pregnant women and non-pregnant women.
- The use of capillary blood for the measurement of haemoglobin concentration in population surveys overestimated the prevalence of anaemia by up to 20.7 percentage points in children and 28.2 percentage points in women.

**What do the new findings imply?**

- The analytic method used to measure haemoglobin concentration is unlikely to introduce variation and influence the diagnosis of anaemia and population prevalence of anaemia.
- Population-based anaemia prevalence statistics derived from capillary haemoglobin concentrations may be inaccurate and lead to incorrect classifications of public health severity.

## Introduction

Anaemia accounted for over 859 age-standardised years lived with disability (per 100 000) in 2013, affecting 1.93 billion people worldwide, with particular burden in low-income and middle-income countries.^1 2^ Anaemia may impair the health, well-being and cognitive functioning of populations,^3 4^ and, if occurring in pregnancy, can cause low birth weight as well as maternal and perinatal mortality.^5 6^ Population estimates of the prevalence of anaemia and programme monitoring of anaemia control interventions, such as micronutrient supplementation and food fortification, depend on haemoglobin concentration measurements in field surveys. Thus, accurate haemoglobin measurement in the field is crucial for improved anaemia control globally. While measurement of haemoglobin on venous blood is considered optimal in clinical practice, collecting venous samples from large numbers of participants (usually young children and women) in field settings where infrastructure is limited is challenging. Therefore, estimates of global anaemia prevalence have been mainly generated from studies that have measured haemoglobin from capillary samples using portable point-of-care devices. For example, the extensive Demographic and Health Survey (DHS) programme utilises capillary haemoglobin concentrations measured using HemoCue devices to estimate population prevalence of anaemia in preschool children and women.^7^

Limited evidence suggests that there may be variation, either systematic or random, in haemoglobin concentration values measured using field-suitable techniques and the true value may be under or overestimated.^8^ Preanalytic factors affecting measurement of haemoglobin concentration are related to the source of blood (capillary vs venous) and this may result in important differences in haemoglobin concentration^8^ that impact on the estimate of anaemia prevalence. Analytic factors, arising due to differences between analytic techniques (ie, different instruments) may also be important.^8^ If there are differences in haemoglobin concentrations between venous and capillary surveys or between instruments, ultimately global estimates of the prevalence of anaemia will be incorrect. It is, therefore, imperative that we understand the magnitude and direction of the variation introduced by salient preanalytic and analytic factors.

We sought to examine and quantify the preanalytic and analytic factors affecting the measurement of haemoglobin concentrations. First, we assessed preanalytic factors using three large field studies to examine differences in haemoglobin concentration between venous-drawn and capillary-drawn blood measured on the same instrument and modelled how differences may affect the estimate of anaemia prevalence in a population. Second, we assessed analytic factors by using data from an international quality assurance programme to assess differences in the measurement of haemoglobin concentration from stabilised blood samples across 16 different commercial haematology instruments.

## Methods

### Preanalytic factors

We examined the difference between haemoglobin concentrations measured using capillary versus venous blood samples in children and pregnant women. For children, we used data from the Benefits and Risks of Iron Interventions in Children (BRISC) trial in Bangladesh and for pregnant women, we used data from the Randomized Controlled Trial of the Effect of Intravenous Iron on Anaemia in Malawian Pregnant Women (REVAMP).

#### BRISC trial

In the BRISC study (ACTRN12617000660381), 3300 children living in Rupganj, Bangladesh, obtained a capillary haemoglobin measurement at 8 months of age (±2 weeks) as part of the trial’s screening procedure.^9 10^ One of the study’s inclusion criteria was that children’s capillary haemoglobin concentration be ≥80 g/L. Those children eligible for enrolment and consenting to participate in the study then obtained a venous haemoglobin measurement an average of 4.2 days after screening (number of days ranged from 0 to 45, 49% of which were ≤1 day). Research assistants were trained in best practice for capillary blood collection, including avoidance of squeezing the finger during collection. The first blood drop was removed and the second drop was used for haemoglobin measurement. Venous blood was collected in an EDTA tube. Immediately after both blood collections, haemoglobin concentration was measured using a HemoCue 301 (HemoCue AB, Angelholm, Sweden). Capillary haemoglobin measurements were performed in the participant’s home. Venous haemoglobin measurements were performed in the participant’s home or in the study centre. The study received ethical approval from the International Center for Diarrheal Diseases, Bangladesh (icddr, b) and Melbourne Health. Data collection used in this analysis took place from July 2017 to February 2019.

#### Randomized Controlled Trial of the Effect of Intravenous Iron on Anaemia in Malawian Pregnant Women

In the REVAMP study (ACTRN12618001268235), pregnant women were enrolled from Blantyre and Zomba districts of Malawi during their second trimester. All women received a capillary and venous haemoglobin measurement at their second study visit, 28 days after enrolment. Measurements were performed within an hour of each other in the study centre. Capillary haemoglobin was measured using the second blood drop with a HemoCue 301(HemoCue AB, Angelholm, Sweden) by trained and qualified phlebotomists. A venous sample was taken using an EDTA tube. Haemoglobin was measured immediately using a HemoCue 301. Samples were stored at 4°C and haemoglobin was then again measured using a Sysmex (Sysmex XP 300 series, Sysmex Corporation, Kobe, Japan) within 8 hours of blood collection. A subsample of 423 women was included in this analysis. Women were included in this analysis if they participated in their second study visit between November 2018 and February 2020. The study received ethical approval from the College of Medicine Research Ethics Committee, Blantyre, Malawi and the Walter and Eliza Hall Institute of Medical Research, Melbourne, Australia.

#### Preanalytic statistical analyses

Analyses were conducted using R V. 3.6.1 (R Foundation for Statistical Computing, Vienna, Austria). Prevalence statistics were derived for anaemia using capillary and venous blood samples. The sensitivity and specificity of anaemia estimates using capillary samples were calculated. Mean haemoglobin concentrations (with SD) were calculated for capillary and venous samples. Pearson’s correlation coefficient was calculated between capillary and venous haemoglobin concentrations, and a kappa coefficient for the diagnosis of anaemia comparing both blood sites. A Bland-Altman analysis was used to examine the agreement between capillary and venous haemoglobin concentration, deriving bias and limits of agreement (LoA) and their respective two-sided 95% CIs. LoAs define where 95% of the differences of the sample between the two methods fall (ie, precision).^11^ For the BRISC participants, sensitivity analyses were run to examine Bland-Altman statistics restricted to those children with ≤1 day versus >1 day between capillary and venous haemoglobin measurement.

#### Modelling-adjusted haemoglobin concentration and anaemia prevalence

Using differences between capillary and venous haemoglobin concentrations in children (derived with BRISC data), in pregnant women (derived with REVAMP data), and in non-pregnant women of reproductive age (using published statistics derived with data from India,^12^ where capillary and venous haemoglobin measurements were performed consecutively on the same day), we adjusted capillary haemoglobin concentrations from the DHS Programme to approximate venous haemoglobin and modelled an adjusted estimate of anaemia prevalence. We used countries’ DHS data where individual haemoglobin concentrations were reported.^13^ For each country, we reported (1) mean haemoglobin concentrations using capillary samples, (2) venous-adjusted haemoglobin concentration after applying the adjustment factor (ie, bias between venous and capillary haemoglobin concentrations), (3) estimated prevalence of anaemia using capillary haemoglobin, (4) venous-adjusted estimated prevalence of anaemia after applying the adjustment factor and (5) percentage point (pp) difference between the estimated prevalence of anaemia using capillary samples and the venous-adjusted prevalence. We supplemented these adjustments using the 95% CI limits around the bias. Finally, we examined how the classification of public health severity of anaemia changed after applying an adjustment for venous haemoglobin concentration.

In children and pregnant women, anaemia was defined as a haemoglobin concentration <110 g/L, mild anaemia as 100–109.9 g/L, moderate anaemia as 70–99.9 g/L and severe anaemia as haemoglobin concentration <70 g/L.^14^ In non-pregnant women of reproductive age, anaemia was defined as a haemoglobin concentration <120 g/L. No adjustment for elevation was required because altitude of residence was below 1000 m. The public health severity of anaemia was defined as severe if the population prevalence was ≥40%, as moderate if the prevalence was ≥20% and<40% and as mild if the prevalence was ≥5% and<20%.^14^

### Analytic factors

To examine analytic differences in the measurement of haemoglobin concentration, we used data from the Royal College of Pathologists of Australasia Quality Assurance Program (RCPAQAP). Ethical approval was not required; all laboratory data were anonymised. Enrolment in an external quality assurance programme is a core requirement of accreditation for clinical laboratories. The RCPAQAP enrolled a total of 1361 distinct laboratories between 2010 and 2019 (between 748 and 890 laboratories per year depending on the year). The majority of laboratories were based in Australia (approximately 60%) and the rest were in New Zealand, Asia, the Pacific and the Middle East. Every month, two stabilised whole blood samples are sent to laboratories enrolled in the programme. Samples are measured on the laboratory haematology analyzer and results are returned to the RCPAQAP. Because samples are highly stable (ie, samples are tested to ensure they withhold degradation associated with transport), differences in the sample haemoglobin concentration measurement can be considered attributable to the instrument itself, that is, analytical factors. The analytical result provided in the QAP data could be a combination of the performance of the laboratory instrument as well as maintenance and calibration, and instruments may not perform well in laboratories that do not maintain them. However, we expect any variation introduced from laboratory quality procedures to be minimal given the laboratories were accredited by the RCPAQAP and underwent regular and rigorous quality assurance. We used data from QA surveys undertaken between January 2010 and October 2019. We used box plots and calculated the mean, SD, minimum and maximum difference between the mean haemoglobin concentration for each instrument and the mean haemoglobin concentration for all remaining instruments, for each quality control sample. We used Bland-Altman plots and statistics to examine the agreement between the mean haemoglobin concentration of each instrument and the mean haemoglobin concentration for all remaining instruments, for each sample. Lin’s concordance correlation coefficients were used to compare mean haemoglobin concentration for each sample measured on different instruments.

Instruments were grouped together by combining models of the same instrument. For all analyses, implausible haemoglobin concentrations <20 and >300 g/L were excluded.

### Participant and public involvement

Participants and the public were not involved in the design of these analyses because the analyses were not part of the original objectives of the studies and were designed post hoc.

## Results

### Preanalytic factors

#### Preschool-age children

In BRISC, 3186 children underwent both capillary and venous measurement at 8 months of age; 114 children (or 3.5%) refused a venous measurement. The prevalence of anaemia using capillary blood was 68.9% (95% CI 67.3 to 70.5) and using venous blood was 44.8% (95% CI 43.1 to 46.5) (online supplemental table 1). The difference in prevalence was largely due to the misdiagnosis of non-anaemic cases (ascertained by venous samples) as mild anaemia when measured using capillary blood (online supplemental table 1). The sensitivity of capillary samples to detect anaemia as defined using venous samples as the gold standard was 89.3%, and the specificity was 47.6%. The kappa coefficient for diagnosis of anaemia was 0.35 accounting for chance.

10.1136/bmjgh-2021-005756.supp1 Supplementary data

**Figure 1:**
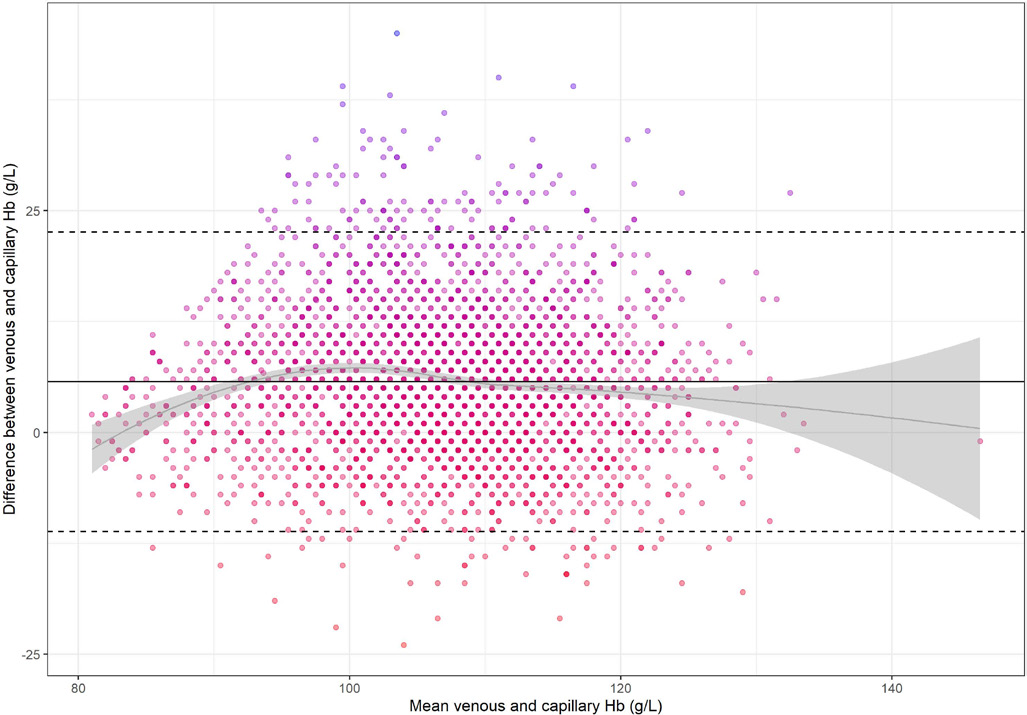
Bland-Altman plot depicting the difference between venous and capillary haemoglobin concentration measured using a HemoCue in Bangladeshi children at 8 months of age (N=3186). The solid black line represents the mean bias 5.7 g/L (95% CI: 5.4 to 6.0). Dotted lines represent the lower and upper limits of agreement [−11.2 g/L (95% CI: –11.7 to –10.6) to 22.6 g/L (95% CI: 22.0 to 23.1)]. The grey curve and grey shaded area represent the bias across haemoglobin concentrations and 95% CI around that bias. Hb, haemoglobin concentration.

Mean±SD haemoglobin concentration using capillary blood was 104±10.1 g/L and using venous blood was 110±9.8 g/L. The Pearson’s correlation between capillary and venous haemoglobin concentration was 0.63 (95% CI 0.60 to 0.65). The difference between venous and capillary haemoglobin concentration was normally distributed and there were no implausible haemoglobin concentrations. Bland-Altman statistics indicate that the bias between venous and capillary haemoglobin concentration was +5.7 g/L (95% CI 5.4 to 6.0). The LoAs between the two preanalytic approaches were −11.2 g/L (95% CI −11.7 to –10.6) to +22.6 g/L (95% CI 22.0 to 23.1) (figure 1). Analyses restricted to children with ≤1 day (49% of participants) and >1 day (51%) between capillary and venous haemoglobin measurement did not meaningfully change the bias (≤1 day: bias +6.3 g/L, LoA −10.7 g/L to +23.3 g/L;>1 day: bias +5.2 g/L, LoA −11.5 g/L to +22.0 g/L).

#### Pregnant women

In REVAMP, the prevalence of anaemia measured using capillary blood was 59.1% (95% CI 54.4 to 63.8) and using venous blood was 65.2% (95% CI 60.7 to 69.7) (online supplemental table 2). The majority of those misdiagnosed were nonanaemic using capillary blood and mildly anaemic using venous blood (online supplemental table 2). The sensitivity of anaemia diagnosis by capillary blood was 80.1% and the specificity was 80.3%. The kappa coefficient for diagnosis of anaemia comparing both blood sites was 0.58 accounting for chance.

**Figure 2:**
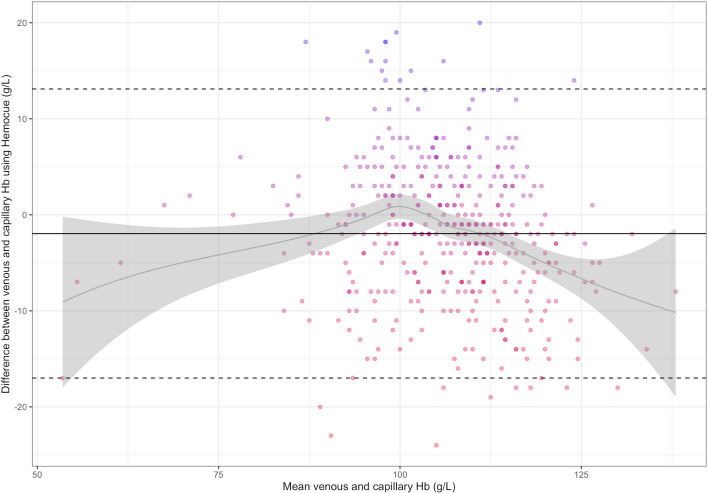
Bland-Altman plot depicting the difference between venous and capillary Hb concentration measured using a HemoCue in Malawian pregnant women (N=423). The solid black line represents the mean bias −2.0 g/L (95% CI: −2.7 to –1.2). Dotted lines represent the lower and upper limits of agreement [−17.0 g/L (95% CI: –17.9 to –16.2) to 13.1 g/L (12.2 to 14.0)]. The grey curve and grey shaded area represent the bias across haemoglobin concentrations and 95% CI around that bias. Hb, haemoglobin concentration.

Mean±SD haemoglobin concentration using capillary blood was 107±11.9 g/L and using venous blood was 105±10.9 g/L. The Pearson’s correlation between capillary and venous haemoglobin concentration was 0.78 (95% CI 0.74 to 0.81). The Bland-Altman statistics indicate that the bias between venous and capillary haemoglobin concentration using the HemoCue was −2.0 g/L (95% CI −2.7 to –1.2) and the LoAs were −17.0 g/L (95% CI −17.9 to –16.2) to 13.1 g/L (12.2 to 14.0) (figure 2). The difference between capillary and venous haemoglobin ranged from −28 g/L to +20 g/L. The Bland-Altman plot indicates that the bias between venous and capillary haemoglobin concentration is U-shaped, with a negative bias at concentrations below 90 g/L and above 110 g/L and a positive bias at concentrations between 90 g/L and 110 g/L (figure 2). The wide LoA indicates large differences between the capillary and venous measurement of haemoglobin concentration.

We observed similar lack of agreement when comparing venous haemoglobin measured using the Sysmex instrument and capillary haemoglobin measured using HemoCue. In this case, the bias was −6.3 g/L (95% CI −7.0 to –5.6) and the LoA was −21.0 g/L (95% CI −21.9 to –20.2) to 8.5 g/L (7.6 to 9.3) (online supplemental figure 1).

Measurement agreement was tighter when comparing venous haemoglobin using Sysmex and venous haemoglobin using HemoCue: the bias was −4.3 g/L (95% CI −4.8 to –3.8) and the LoA was −13.9 g/L (95% CI −14.4 to –13.3) to 5.3 g/L (4.7 to 5.8) (online supplemental figure 2).

#### Modelling venous-adjusted haemoglobin concentration using DHS data

Using DHS data for children from 54 countries, venous-adjusted haemoglobin concentration was approximated by adding 5.7 g/L (the bias between venous and capillary haemoglobin found in BRISC) to the reported capillary haemoglobin concentration. Using DHS data from non-pregnant women of reproductive age from 51 countries, venous-adjusted haemoglobin concentration was approximated by adding 8.8 g/L (the bias reported in a previous study of Indian non-pregnant women^12^ to the reported capillary haemoglobin concentration). Venous-adjusted anaemia prevalence was calculated using venous-adjusted haemoglobin concentrations. Due to the inconsistent bias observed in our data in pregnant women (ie, U-shaped curve for the bias between venous and capillary haemoglobin concentration across haemoglobin concentration (figure 2)), we did not apply a venous adjustment to haemoglobin concentration data in this population.

**Figure 3:**
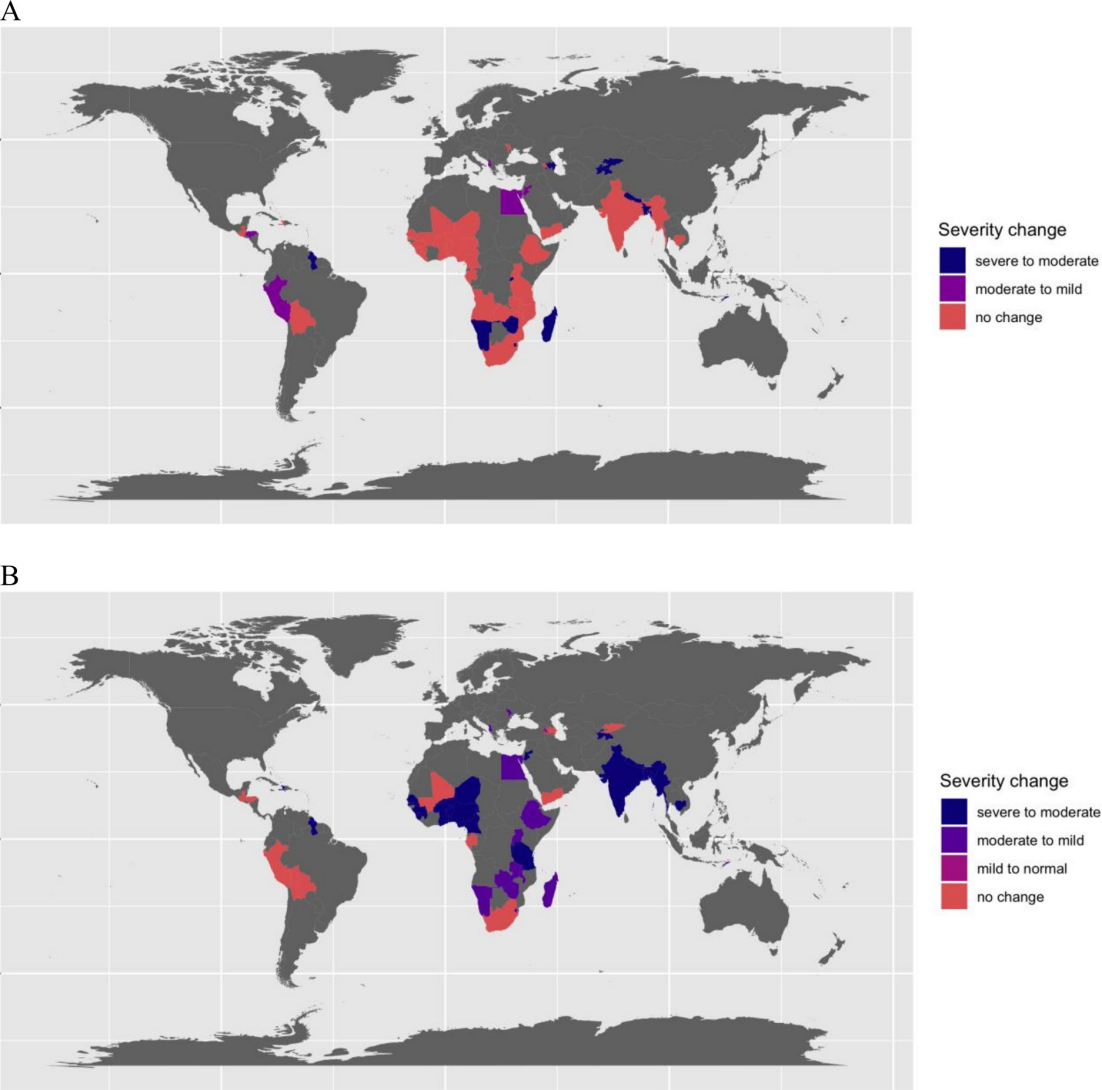
Anaemia public health severity level change after venous adjustment to haemoglobin concentration in preschool-age children (A) and in non-pregnant women of reproductive age (B). The public health severity of anaemia was defined as severe if the population prevalence was ≥40%, as moderate if the prevalence was ≥20% and <40% and as mild if the prevalence was ≥5% and<20%.

In children, the mean±SD haemoglobin concentration and prevalence of anaemia using capillary samples ranged from 89±18.1 g/L and 88% anaemia in Yemen to 120±12.2 g/L and 18.1% anaemia in Armenia. After venous adjustment, the prevalence of anaemia ranged from 80.6% in Yemen to 8.3% in Armenia. The pp difference in prevalence of anaemia after venous adjustment ranged from −7.4 pp in Yemen to −20.7 pp in Myanmar (online supplemental table 3). Of the 54 DHS surveys included in the analysis of children, 35 (64.8%) did not change their public health severity level for anaemia after venous adjustment, 5 (9.3%) changed from moderate to mild severity and 14 (25.9%) changed from severe to moderate (figure 3).

In non-pregnant women of reproductive age, the mean±SD haemoglobin concentration and prevalence of anaemia using capillary samples ranged from 107±19.2 g/L and 72.7% anaemia in Yemen to 134±14.2 g/L and 14.4% anaemia in Guatemala. After venous adjustment, the prevalence of anaemia ranged from 54.5% in Yemen to 4.4% in Armenia. The pp difference in prevalence of anaemia after venous adjustment ranged from −8.8 pp in Guatemala to −28.2 pp in the Maldives (online supplemental table 4). Of the 51 DHS surveys included in the analysis of non-pregnant women, 10 (19.6%) did not change their public health severity level for anaemia, 1 (2%) changed from mild to normal, 14 (27.5%) changed from moderate to mild and 26 (51.0%) changed from severe to moderate (figure 3).

In children and women, venous adjustment using the 95% CI limits around the bias (ie, 5.4 and 6.0 g/L in children; 8.4 and 9.3 g/L in non-pregnant women) resulted in the same estimated prevalence of anaemia when compared with using the bias (ie, 5.6 g/L in children; 8.8 g/L in non-pregnant women).

### Analytic factors

A total of 38 haematology analyzer models were collapsed into 16 instrument categories. Data compiled from 2010 to 2019 yielded between 19 and 172 487 individual sample measurements depending on the instrument. The most common instruments contributing to the measurements in the QAP throughout the entire time period were the Sysmex (1 72 487 samples completed across all laboratories over the 10-year period), Beckman Coulter (57 984 samples) and Cell Dyne (27 566 samples). The number of individual instruments varied year to year, but in October 2019 (the most recent dataset), out of the 1271 instruments enrolled in the QAP, 72.0% were Sysmex, 13.3% were Beckman Coulter, 6.6% were Cell Dyne, 2.4% were Siemens, 1.9% were Nihon Khoden, 1.9% were Mindray and the remaining instruments each represented less than 1%. When like-instruments were grouped across laboratories (for the Bland-Altman analyses) and each instrument’s QC sample measurements were averaged, grouped instrument–sample pairs ranged from 10 to 240. Some instruments did not contribute a measurement at every timepoint because they either were introduced or removed by a laboratory over the time period.

All concordance correlation coefficients were ≥0.98, indicating substantial concordance between the instruments.

**Figure 4:**
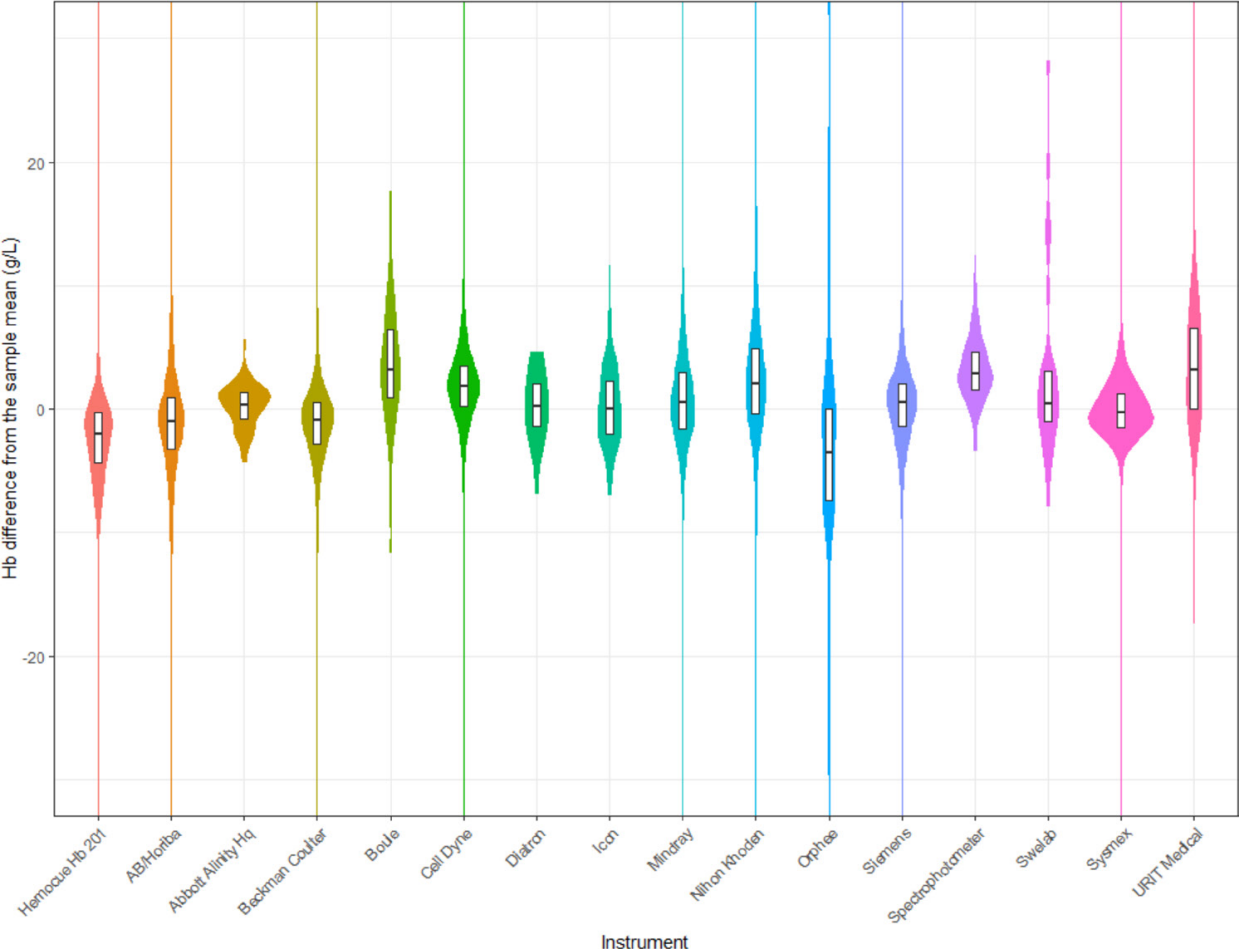
Violin plot of the difference between each individual Hb measurement minus the mean Hb concentration for all remaining instruments in that quality control sample, by instrument. Box plot within each violin plot represents the median and IQR. All remaining instruments refer to all instruments excluding the one being examined. Hb, haemoglobin concentration.

The mean haemoglobin concentration difference from the sample mean, excluding that instrument, was <4 g/L for all instruments. The mean±SD difference ranged from −3.8±6.7 g/L for Orphee instruments to 3.6±4.2 g/L for Boule instruments (figure 4, (online supplemental table 5).

**Figure 5:**
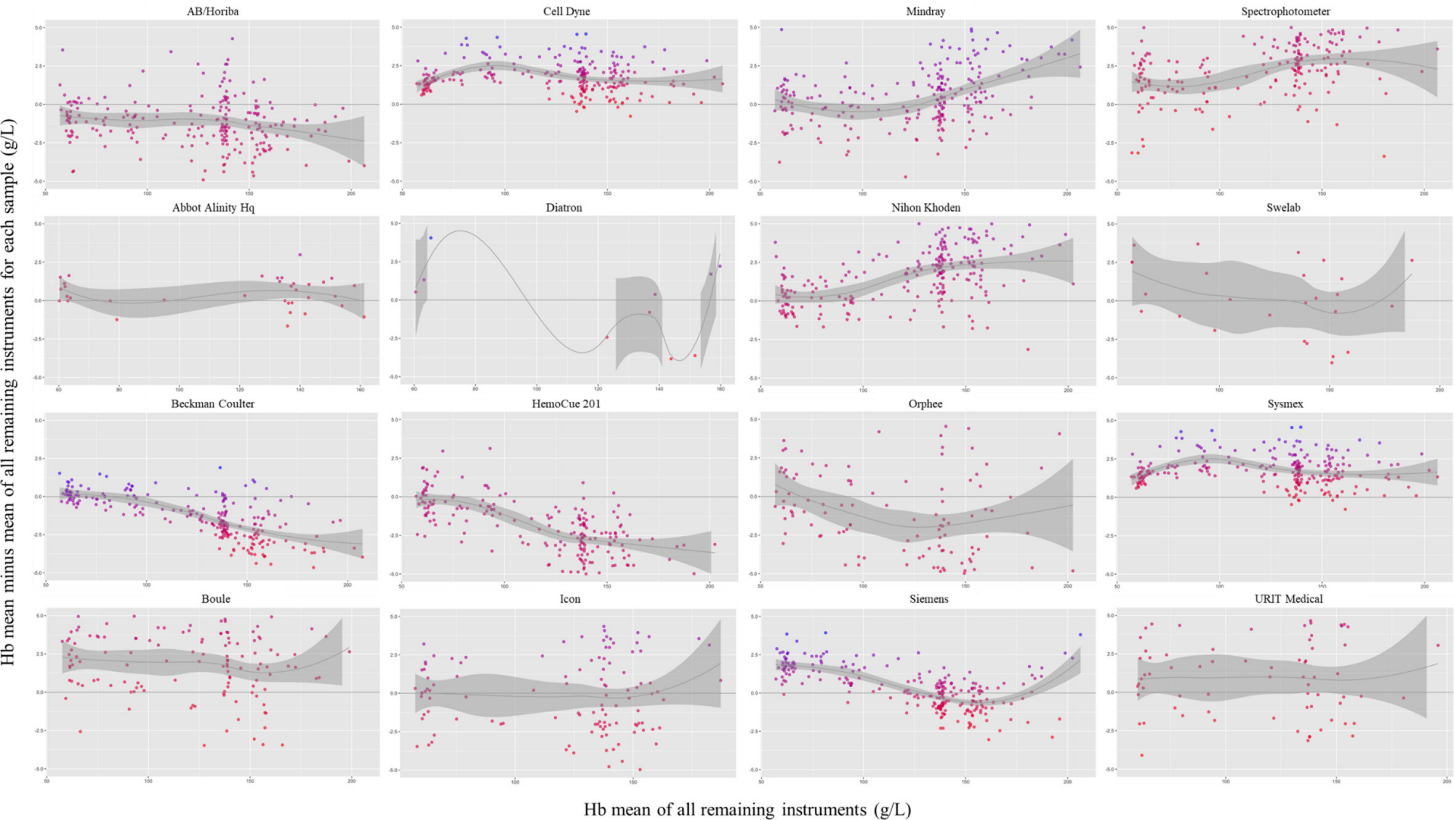
Panel of Bland-Altman plots depicting the difference between the mean haemoglobin concentration for each instrument and the mean Hb concentration for all remaining instruments for each quality control sample, by instrument. Each point represents a quality control sample (the mean Hb concentration in a sample for all like-instruments minus the mean Hb concentration for all remaining instruments). The y-axis was trimmed from −5 to 5 g/L despite few outliers being outside this range. All remaining instruments refer to all instruments excluding the one being examined. Hb, haemoglobin.

Bland-Altman plots indicate that, for each sample, the difference between the mean haemoglobin concentration for each instrument and the mean haemoglobin concentration for all remaining instruments was minimal (figure 5). The mean±SD difference ranged from −3.3±6.3 g/L for Orphee instruments to 3.6±3.3 g/L for Boule instruments. Among the most commonly used instruments, Sysmex had the smallest bias (−0.1 g/L), followed by Beckman Coulter (−1.3 g/L) and Cell Dyne (1.7 g/L). The bias for the HemoCue was −2.5 g/L (online supplemental table 6).

## Discussion

Accurate and precise measurement of haemoglobin concentration is critical for estimation of population-level anaemia prevalence, which forms the basis for defining nutrition policy choices^15^ and monitoring programmes. We examined the preanalytic and analytic factors that may affect haemoglobin concentration. In preschool children, though not in pregnant women, a significant systematic difference, or bias, was observed between capillary-drawn and venous-drawn haemoglobin concentration. Moreover, in all populations analysed, there was considerable imprecision when comparing capillary to venous haemoglobin measured on the same instrument. In contrast, when we used a large quality assurance programme to assess analytic factors, we found that essentially all current instruments for haemoglobin measurement performed similarly, and, thus, analytic factors are unlikely to account for substantial variation in haemoglobin concentrations. These data indicate that accurate estimation of anaemia prevalence from large field studies using capillary samples may not be achievable, regardless of the specific instrument used.

We observed that the bias between capillary and venous haemoglobin in our cohort of preschool South Asian children (difference of 5.7 g/L) was similar to previously published differences seen in non-pregnant women of reproductive age living in India (8.8 g/L).^8^ In contrast, in pregnant women in Malawi, the mean difference between capillary and venous haemoglobin varied over the range of haemoglobin concentrations. This could be due to haemodilution during pregnancy, but remains to be further investigated.^16^ After modelling a venous adjustment to capillary haemoglobin concentrations in DHS data from across the world, the estimated prevalence of anaemia fell by up to 20.7 pps in children and 28.2 pps in non-pregnant women. Particularly, in women, venous adjustment resulted in meaningful reductions in countries’ classification of anaemia severity. These findings suggest that preanalytic factors may significantly affect the prevalence of anaemia and how a country perceives its public health severity. This has important implications for anaemia reduction strategies and monitoring of population anaemia prevalence over time. A limitation of this modelling exercise was that haemoglobin adjustments were based on the bias between capillary and venous haemoglobin concentrations observed in only two studies. Furthermore, our data did not suggest clinically meaningful differences in the SD of capillary and venous haemoglobin; therefore, we did not adjust the SD of venous haemoglobin. However, the large population-based studies from which these bias estimates were derived used rigorous standards for haemoglobin measurement and analysis. Overall, the reductions in anaemia prevalence estimates demonstrate how influential even small differences in haemoglobin concentrations introduced by preanalytic factors can be.

We analysed data from 1361 laboratories enrolled in an international quality assurance programme to assess 16 haematology analysers. In general, the difference in haemoglobin concentration between venous samples analysed on different instruments was minimal and not clinically meaningful. Other studies performing pairwise comparisons between instruments have shown similar findings. For instance, in a study of Bangladeshi children, non-pregnant and pregnant women, venous haemoglobin concentrations measured on a HemoCue 201 were similar to the same samples measured on a Sysmex (mean difference of 0.3 g/L).^17^ In a study of male and female blood donors in India, the difference between haemoglobin measurements using a HemoCue 301 versus HemoCue 201+ ranged from 2.54 g/L when using the second drop of blood to 3.55 g/L using the first drop of blood.^18^ A recent review indicated that out of 11 studies measuring venous haemoglobin using a HemoCue 201 compared with an automated haematology analyser, the difference ranged from 0.2 g/L to 16.0 g/L; the majority was within the allowable degree of variation (within 7% bias).^19^ Our analysis of the large RCPAQAP provides reassurance that in the context of laboratories participating in QAP programmes, the choice of haematology analysers, including the HemoCue, is unlikely to explain substantial variation. Results from the analysis of RCPAQAP data would extend to haematology analysers and point-of-care devices managed within a comprehensive quality assurance programme; if devices are not well managed, measurements could be affected by external factors such as instrument calibration, reagent storage conditions and humidity.

Our results indicate that there is substantial bias and considerable imprecision when comparing capillary and venous sampling for the measurement of haemoglobin. This creates a major impediment for field-based population surveys, which typically use capillary samples to measure haemoglobin^7^ and derive the prevalence of anaemia. This information is directly used to inform population-level interventions such as iron or micronutrient supplementation and food fortification.^20^ Our data suggest that estimates of the prevalence of anaemia would change by collecting venous rather than capillary samples and improve by ensuring that these are measured using a quality laboratory or using a high-quality point of care device such as the HemoCue 201+. Further research into improving preanalytic factors affecting capillary haemoglobin measurements, such as using pooled blood rather than single drops^19^ and variation across device operators,^21^ is also warranted.

Analytic variation in haemoglobin measurements is unlikely to influence interpretation of a diagnosis of anaemia in the clinical or public health setting. However, our data clearly indicate that capillary and venous samples cannot be used interchangeably for the measurement of haemoglobin concentration and estimation of anaemia prevalence given the bias and imprecision when comparing the two. These findings call the global estimates of anaemia prevalence, which have been predominantly generated using capillary measurements, into doubt. Future surveys should strongly consider using venous samples, even if this requires smaller samples sizes.

## Data Availability

Data are available upon request. Data will be available upon appropriate request from the authors, following database lock, unblinding and publication of the parent clinical trials from which data from this study is derived.
