# Supplementary material for: Preanalytic and analytic factors affecting the measurement of haemoglobin concentration: impact on global estimates of anaemia prevalence
Source: BMJ Glob Health. 2021 Jul 30;6(7):e005756. doi: 10.1136/bmjgh-2021-005756 (PMC8327809; doi:10.1136/bmjgh-2021-005756)
Supplement: Supplementary data [file bmjgh-2021-005756supp001.pdf]

Online Supplementary Material

Supplemental Table 1: Anemia prevalence using capillary vs venous blood samples in 3186 children at 8 months of age[1]

Dichotomous anemia

			Venous sample: Any anemia		
			**No**	**Yes**	**Total**
**Capillary sample**	**Any anemia**	**No**	26.3 (838)	4.8 (152)	31.1 (990)
		**Yes**	28.9 (921)	40.0 (1275)	68.9 (2196)
		**Total**	55.2 (1759)	44.8 (1427)	100 (3186)

Categorical anemia

		Venous sample			
		**No anemia**	**Mild anemia**	**Moderate anemia**	**Total**
**Capillary sample**	**No anemia**	26.3 (838)	4.5 (143)	0.3 (9)	31.1 (990)
	**Mild anemia**	21.3 (679)	14.1 (450)	2.7 (85)	38.1 (1214)
	**Moderate anemia**	7.6 (242)	12.3 (392)	10.9 (348)	30.8 (982)
	**Total**	55.2 (1759)	30.9 (985)	13.9 (442)	100 (3186)

[1]Values are % (N). Anemia was defined as a hemoglobin concentration (hb) <110 g/L, mild anemia as 100-109.9 g/L, and moderate anemia as 70-99.9 g/L. Children with severe anemia (hb<70 g/L) were excluded from the study.

Supplemental Table 2: Anemia prevalence using capillary vs venous blood samples in 423 pregnant women[1]

Dichotomous anemia

			Venous sample: Any anemia		
			No	Yes	Total
Capillary sample	Any anemia	No	27.9 (118)	13.0 (55)	40.9 (173)
		Yes	6.9 (29)	52.2 (221)	59.1 (250)
		Total	34.8 (147)	65.2 (276)	100 (423)

Categorical anemia

		Venous sample				
		No anemia	Mild anemia	Moderate anemia	Severe anemia	Total
Capillary sample	No anemia	29.1 (123)	14.2 (60)	0.9 (4)	0	44.2 (187)
	Mild anemia	5.2 (22)	18.4 (78)	8.0 (34)	0	31.7 (134)
	Moderate anemia	0.5 (2)	8.3 (35)	14.4 (61)	0	23.2 (98)
	Severe anemia	0	0	0	0.9 (4)	0.9 (4)
	Total	34.8 (147)	40.9 (173)	23.4 (99)	0.9 (4)	100 (423)

[1]Values are % (N). Anemia was defined as a hemoglobin concentration (hb) <110 g/L, mild anemia as 100-109.9 g/L, moderate anemia as 70-99.9 g/L, and severe anemia as hb < 70 g/L.

Supplemental Table 3: Capillary and venous-adjusted hemoglobin concentration and anemia prevalence in preschool-age children from Demographic and Health Surveys[1]

			Capillary			Venous-adjusted			
**Country**	**Survey year**	**N**	**Mean Hb (g/L)**	**SD Hb (g/L)**	**Anemia %**	**Mean Hb (g/L)**	**SD Hb (g/L)**	**Anemia %**	**Percentage point difference between capillary and venous-adjusted anemia prevalence**
Albania	2017	2042	116	12.2	27.6	122	12.2	13.7	13.9
Angola	2015	6767	104	14.6	67.7	110	14.6	49.0	18.7
Armenia	2015	1369	120	12.2	18.1	126	12.2	8.3	9.8
Azerbaijan	2006	1840	112	12.8	41.4	118	12.8	26.1	15.3
Bangladesh	2011	2361	108	12.5	54.9	114	12.5	34.7	20.2
Benin	2017	6202	101	15.1	73.8	106	15.1	57.4	16.4
Bolivia	2008	2522	104	16.3	62.9	110	16.3	48.0	14.9
Burkina Faso	2016	5557	99	15.6	76.7	105	15.6	63.5	13.2
Burundi	2016	5729	104	16.1	63.6	109	16.1	48.0	15.6
Cambodia	2014	4468	107	12.8	59.0	113	12.8	40.0	19.0
Cameroon	2011	5386	105	15.3	62.8	111	15.3	47.1	15.7
Democratic Republic of the Congo	2013	8167	104	17.0	61.6	110	17.0	46.4	15.2
Ivory Coast	2011	3371	98.7	15.6	76.8	104	15.6	63.1	13.7
Egypt	2014	4651	115	11.8	31.1	121	11.8	16.6	14.5
Ethiopia	2016	8437	105	17.6	59.9	111	17.6	44.0	15.9
Gabon	2012	3798	105	14.2	63.6	110	14.2	44.3	19.3
Gambia	2013	3299	99	15.9	75.4	105	15.9	61.9	13.5
Ghana	2016	3046	103	14.9	65.7	109	14.9	47.9	17.8
Guatemala	2014	10856	114	13.2	35.5	120	13.2	20.7	14.8
Guinea	2018	3604	100	13.9	77.4	106	13.9	60.9	16.5
Guyana	2009	1613	112	13.5	41.5	118	13.5	27.6	13.9
Haiti	2016	6059	103	14.0	69.3	109	14.0	51.5	17.8
Honduras	2011	9381	115	12.5	32.5	121	12.5	17.9	14.6
India	2015	216863	106	15.3	61.1	111	15.3	45.6	15.5
Jordan	2017	8762	115	12.5	34.6	120	12.5	19.6	15.0
Kyrgyzstan	2012	4178	111	15.4	45.7	116	15.4	29.5	16.2
Lesotho	2014	1726	109	16.4	53.0	115	16.4	37.7	15.3
Liberia	2016	2792	99	14.6	79.3	104	14.6	65.0	14.3
Madagascar	2016	7006	110	14.2	48.1	116	14.2	32.8	15.3
Malawi	2017	2296	104	15.1	64.0	110	15.1	49.9	14.1
Maldives	2016	2058	109	12.9	53.1	115	12.9	33.8	19.3
Mali	2018	4334	95.4	15.8	83.8	101	15.8	70.6	13.2
Moldova	2005	1326	114	11.5	35.6	119	11.5	20.2	15.4
Mozambique	2018	4336	96	16.4	82.3	102	16.4	69.3	13.0
Myanmar	2015	3926	106	13.7	61.4	112	13.7	40.7	20.7
Namibia	2013	2312	110	14.5	49.9	115	14.5	32.9	17.0
Nepal	2016	2177	108	13.4	56.5	113	13.4	37.9	18.6
Niger	2012	4799	99.9	15.1	76.1	106	15.1	61.1	15.0
Nigeria	2018	11206	102	15.6	70.4	107	15.6	54.0	16.4
Peru	2012	8646	114	12.7	35.0	120	12.7	19.6	15.4
Rwanda	2010	4009	113	13.7	40.9	119	13.7	23.6	17.3
Sao Tome and Principe	2008	1766	105	12.8	65.4	111	12.8	45.4	20.0
Senegal	2017	10845	101	15.0	73.4	107	15.0	56.8	16.6

Sierra Leone	2016	6656	98	15.8	78.0	104	15.8	63.8	14.2
South Africa	2016	1136	104	15.8	64.2	110	15.8	50.2	14.0
Swaziland	2006	2521	112	15.2	43.2	118	15.2	28.0	15.2
Tajikistan	2017	5461	111	13.8	44.9	117	13.8	28.4	16.5
Tanzania	2017	7200	106	14.3	61.3	112	14.3	43.6	17.7
Timor-Leste	2016	2031	111	12.2	46.1	117	12.2	26.2	19.9
Togo	2017	3206	99	15.3	77.5	105	15.3	61.4	16.1
Uganda	2016	4755	107	16.0	55.6	112	16.0	40.1	15.5
Yemen	2013	3856	89	18.1	88.0	95	18.1	80.6	7.4
Zambia	2018	8607	106	14.3	61.3	112	14.3	42.5	18.8
Zimbabwe	2015	5208	113	13.3	40.4	119	13.3	23.3	17.1

[1]For all country datasets, minimum age was 6 months and maximum age was 59 months. Venous-adjusted hemoglobin concentration was calculated by adding 5.7 g/L to the reported capillary hemoglobin concentration. Hb, hemoglobin concentration; SD, standard deviation.

Supplemental Table 4: Capillary and venous-adjusted hemoglobin concentration and anemia prevalence in non-pregnant women of reproductive age from Demographic and Health Surveys[1]

			Capillary			Venous-adjusted			
**Country**	**Survey year**	**N**	**Mean Hb (g/L)**	**SD Hb (g/L)**	**Anemia %**	**Mean Hb (g/L)**	**SD Hb (g/L)**	**Anemia %**	**Percentage point difference between capillary and venous-adjusted anemia prevalence**
Albania	2017	10193	127	13.8	24.8	136	13.8	10.0	14.8
Armenia	2015	5644	132	12.8	15.4	141	12.8	4.4	11.0
Azerbaijan	2006	7834	122	15.7	38.8	131	15.7	20.3	18.5
Bangladesh	2011	5342	122	13.6	44.6	130	13.6	20.6	24.0
Benin	2017	7129	116	15.5	59.4	125	15.5	34.9	24.5
Bolivia	2008	5398	123	16.4	39.5	132	16.4	20.9	18.6
Burkina Faso	2010	7556	119	17.5	50.0	127	17.5	30.3	19.7
Burundi	2016	7875	122	16.3	41.0	131	16.3	22.6	18.4
Cambodia	2014	10731	120	13.3	48.4	129	13.3	22.9	25.5
Cameroon	2011	7066	123	16.4	41.0	131	16.4	21.2	19.8
Democratic Republic of the Congo	2013	8125	123	15.9	40.1	132	15.9	21.1	19.0
Ivory Coast	2011	4196	117	16.4	56.5	126	16.4	33.5	23.0
Egypt	2014	6464	126	11.2	29.9	135	11.2	9.0	20.9
Ethiopia	2016	13436	129	17.8	24.9	138	17.8	12.6	12.3
Gabon	2012	4906	114	16.0	64.2	123	16.0	40.0	24.2
Gambia	2013	4110	114	17.4	62.7	123	17.4	37.7	25.0
Ghana	2014	4352	120	14.9	45.4	129	14.9	24.5	20.9
Guatemala	2014	24038	134	14.2	14.4	142	14.2	5.6	8.8
Guinea	2018	4203	119	16.1	50.4	128	16.1	27.0	23.4
Guyana	2009	4384	122	15.8	40.2	131	15.8	20.3	19.9
Haiti	2016	9003	118	17.5	51.5	127	17.5	29.4	22.1
Honduras	2011	20385	132	13.6	16.6	141	13.6	6.2	10.4
India	2015	653022	117	16.4	55.8	126	16.4	32.5	23.3
Jordan	2017	6323	120	14.8	47.2	129	14.8	24.3	22.9
Kyrgyzstan	2012	7469	123	16.8	37.8	131	16.8	20.7	17.1
Lesotho	2014	3210	128	18.3	28.8	137	18.3	16.2	12.6
Madagascar	2008	7580	124	15.4	38.1	133	15.4	18.4	19.7
Malawi	2015	7338	125	16.7	34.3	134	16.7	17.3	17.0
Maldives	2016	6611	114	13.7	66.3	123	13.7	38.1	28.2
Mali	2018	4528	113	17.0	65.1	122	17.0	42.5	22.6
Moldova	2005	6906	126	13.8	30.3	134	13.8	13.1	17.2
Myanmar	2015	12019	120	15.3	49.0	129	15.3	24.7	24.3
Namibia	2013	4052	131	16.2	22.2	140	16.2	10.8	11.4
Nepal	2016	6134	122	14.6	43.6	131	14.6	21.6	22.0
Niger	2012	4368	120	16.1	47.3	129	16.1	24.9	22.4
Nigeria	2018	13220	116	14.9	60.4	125	14.9	34.8	25.6
Peru	2012	22447	130	13.4	18.9	139	13.4	7.2	11.7
Rwanda	2014	6212	131	15.0	20.5	140	15.0	8.8	11.7
Sao Tome and Principe	2008	2318	121	15.1	46.1	130	15.1	23.6	22.5
Senegal	2017	7297	117	15.9	56.3	126	15.9	31.6	24.7
Sierra Leone	2008	3096	120	16.7	47.1	129	16.7	28.4	18.7

South Africa	2016	2866	125	18.9	34.9	134	18.9	20.3	14.6
Swaziland	2006	4343	127	17.0	30.8	136	17.0	16.2	14.6
Tajikistan	2017	9882	121	15.1	44.2	129	15.1	21.9	22.3
Tanzania	2015	11977	120	17.0	46.0	129	17.0	25.5	20.5
Timor-Leste	2016	4036	127	13.3	26.2	135	13.3	10.3	15.9
Togo	2017	4193	118	15.2	54.6	126	15.2	30.4	24.2
Uganda	2016	5391	126	15.8	33.4	135	15.8	16.2	17.2
Yemen	2013	4294	107	19.2	72.7	117	19.2	54.5	18.2
Zambia	2018	12153	126	16.9	32.7	135	16.9	16.4	16.3
Zimbabwe	2015	8701	128	17.4	28.9	136	17.4	14.8	14.1

[1]For all country datasets, minimum age was 15 years and maximum age was 49 years. Venous-adjusted hemoglobin concentration was calculated by adding 8.8 g/L to the reported capillary hemoglobin concentration. Hb, hemoglobin concentration; SD, standard deviation.

Supplemental Table 5: Difference from the sample mean hemoglobin concentration for all remaining instrument[1]

Instrument group	N	Mean (g/L)	SD (g/L)	Min (g/L)	Max (g/L)
AB/Horiba	2292	-1.3	7.4	-98	151
Abbott Alinity Hq	88	0.2	1.7	-4	6
Beckman Coulter	57984	-1.2	3.6	-116	126
Boule	332	3.6	4.2	-12	18
Cell Dyne	27566	1.8	3.8	-167	78
Diatron	19	0.1	2.9	-7	5
HemoCue 201	1194	-2.3	6.0	-77	83
Icon	184	0.1	3.2	-7	12
Mindray	3237	0.8	7.1	-167	108
Nihon Khoden	8425	2.2	7.2	-167	81
Orphee	308	-3.8	6.7	-44	35
Siemens	9609	0.3	4.8	-106	126
Spectrophotometer	240	3.1	2.5	-3	12
Swelab	34	2.7	7.5	-8	28
Sysmex	172487	-0.1	3.5	-183	189
URIT Medical	327	3.6	5.8	-17	52

[1]All remaining instruments refers to all instruments excluding the one being examined.

Supplemental Table 6: Summary statistics for Bland Altman plots; mean hemoglobin concentration for each instrument minus the mean hemoglobin concentration for all remaining instruments, for each sample[1]

Instrument group	N	Mean (g/L)	SD (g/L)	Min (g/L)	Max (g/L)
AB/Horiba	236	-1.3	2.5	-12.8	11.6
Abbott Alinity Hq	36	0.6	1.3	-1.7	5.6
Beckman Coulter	240	-1.3	1.4	-5.1	1.9
Boule	186	3.6	3.3	-3.5	13.3
Cell Dyne	240	1.7	1.0	-0.8	4.6
Diatron	10	-0.1	2.6	-3.8	4.1
HemoCue 201	240	-2.5	3.4	-22.8	22.8
Icon	118	-0.1	2.8	-6.6	8.8
Mindray	240	0.7	2.2	-9.6	7.4
Nihon Khoden	240	1.8	2.2	-9.4	12.2
Orphee	172	-3.3	6.3	-25.3	35.0
Siemens	240	0.3	1.4	-3.0	3.9
Spectrophotometer	240	3.1	2.5	-3.4	12.5
Swelab	34	2.7	7.5	-7.8	28.2
Sysmex	240	-0.1	1.2	-3.3	2.5
URIT Medical	112	3.5	4.1	-4.1	14.5

[1]All remaining instruments refers to all instruments excluding the one being examined.

Supplemental Figure 1: Bland-Altman plot depicting the difference between venous hemoglobin concentration measured using Sysmex and capillary hemoglobin concentration measured using a HemoCue in Malawian pregnant women (N=423)

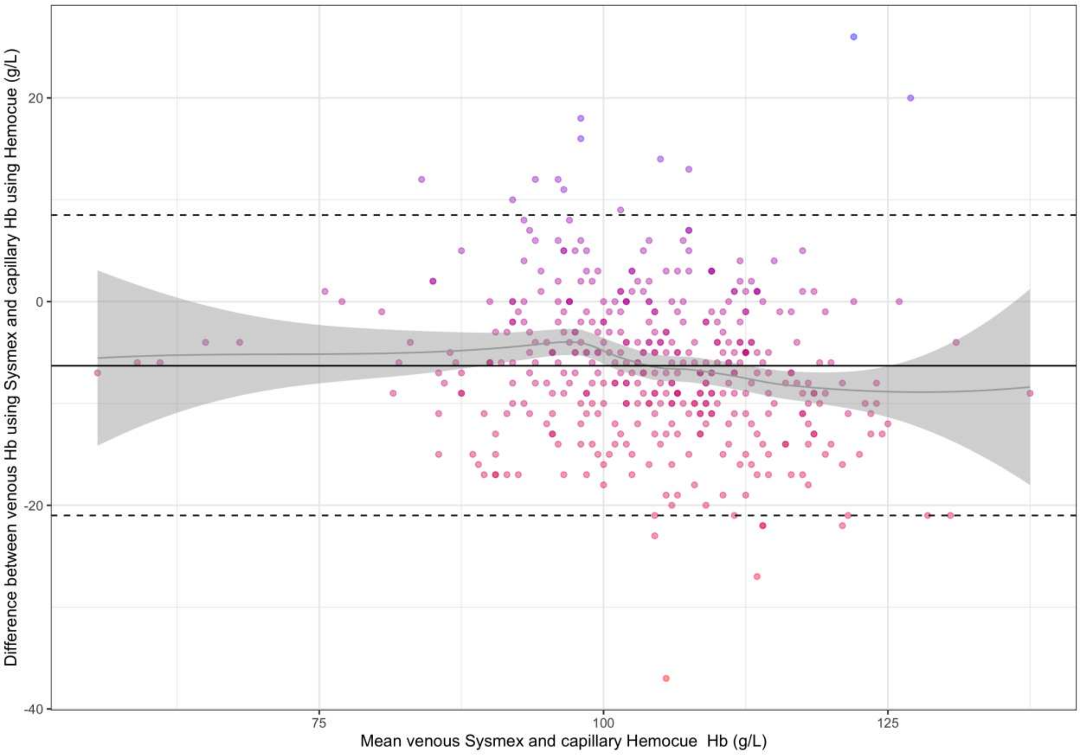

The solid black line represents the mean bias -6.3 g/L (95% CI: -7.0, -5.6). Dotted lines represent the lower and upper limits of agreement [-21.0 g/L (95% CI: -21.9, -20.2) to 8.5 g/L (7.6, 9.3)]. The grey curve and grey shaded area represent the bias across hemoglobin concentrations and 95% CI around that bias. Hb, hemoglobin concentration.

Supplemental Figure 2: Bland-Altman plot depicting the difference between venous hemoglobin concentration measured using Sysmex and venous hemoglobin concentration measured using a HemoCue in Malawian pregnant women (N=423)

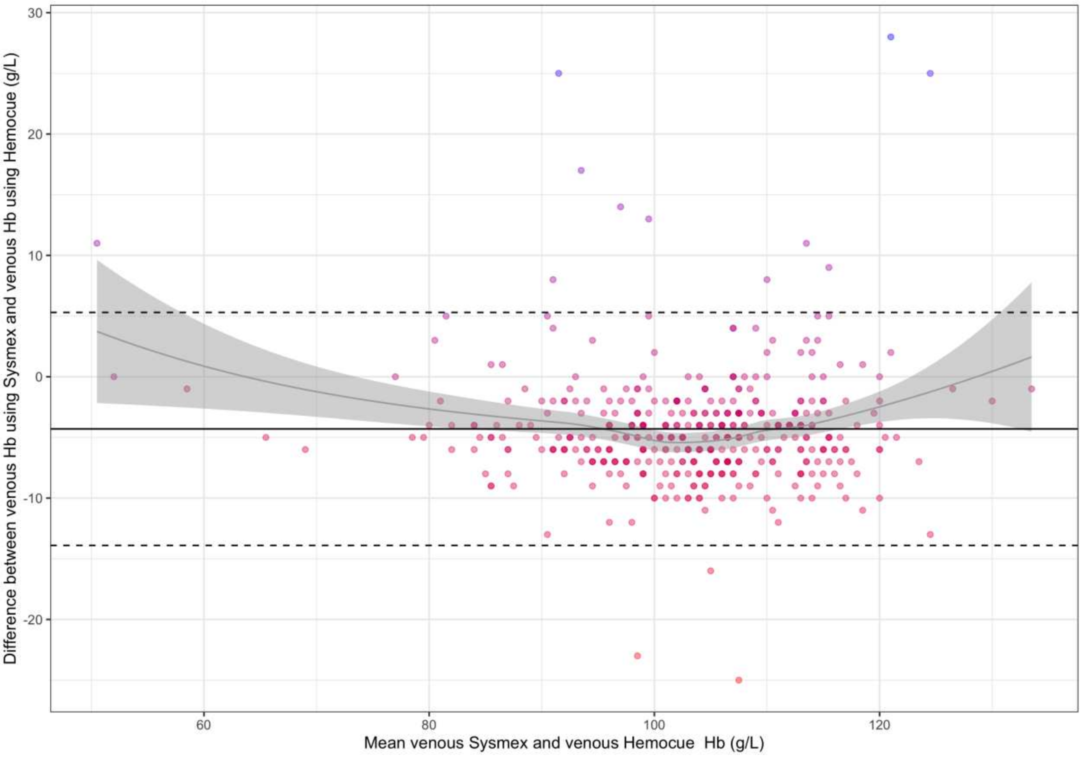

The solid black line represents the mean bias -4.3 g/L (95% CI: -4.8, -3.8). Dotted lines represent the lower and upper limits of agreement [-13.9 g/L (95% CI: -14.4, -13.3) to 5.3 g/L (4.7, 5.8)]. The grey curve and grey shaded area represent the bias across hemoglobin concentrations and 95% CI around that bias. Hb, hemoglobin concentration.
